# Polyphosphate-dependent nicotinamide adenine dinucleotide (NAD) kinase: A novel missing link in human mitochondria

**DOI:** 10.2183/pjab.97.024

**Published:** 2021-10-11

**Authors:** Kousaku MURATA

**Affiliations:** *1 Professor Emeritus, Kyoto University, Kyoto, Japan.

**Keywords:** polyphosphate, NAD^+^, NADP^+^, NAD kinase, NAD kinase gene, human mitochondrial NAD kinase

## Abstract

Polyphosphate [poly(P)] is described as a homopolymer of inorganic phosphates. Nicotinamide adenine dinucleotide kinase (NAD kinase) catalyzes the phosphorylation of NAD^+^ to NADP^+^ in the presence of ATP (ATP-NAD kinase). Novel NAD kinase that explicitly phosphorylates NAD^+^ to NADP^+^ using poly(P), besides ATP [ATP/poly(P)-NAD kinase], was found in bacteria, in particular, Gram-positive bacteria, and the gene encoding ATP/poly(P)-NAD kinase was also newly identified in *Mycobacterium tuberculosis* H37Rv. Both NAD kinases required multi-homopolymeric structures for activity expression. The enzymatic and genetic results, combined with their primary and tertiary structures, have led to the discovery of a long-awaited human mitochondrial NAD kinase. This discovery showed that the NAD kinase is a bacterial type of ATP/poly(P)-NAD kinase. These pioneering findings, *i.e.*, ATP/poly(P)-NAD kinase, NAD kinase gene, and human mitochondrial NAD kinase, have significantly enhanced research on the biochemistry, molecular biology, and evolutionary biology of NAD kinase, mitochondria, and poly(P), including some biotechnological knowledge applicable to NADP^+^ production.

## Introduction

Polyphosphate [poly(P)] is a negatively charged homopolymer with a few to several hundred residues of orthophosphate. A number of physicochemical functions such as storage for energy and phosphate, chelator for divalent cations, and regulator of pH have been assigned to polymer; thus, due to these unique properties, poly(P) has been widely used in many industries.^1,2)^

Poly(P) was first identified in yeast cells more than 100 years ago^3)^ and is now considered to occur in representatives of all kingdoms of living organisms.^4)^ Poly(P) has been believed to be synthesized as an energy-rich compound on the prebiotic earth. However, its importance had been downplayed as a “fossil molecule” or “bioenergy fossil”, because the importance of poly(P) as an energy storage molecule decreased during evolution from prokaryotes to eukaryotes.^5)^ Although the enzymes responsible for poly(P) metabolism can be found in prokaryotes, but not in eukaryotes, they are often scarce. These enzymes are as follows: poly(P) kinase,^6)^ poly(P)-dependent glucokinase,^7)^ poly(P)-dependent adenosine 5′-monophosphate (AMP)-phosphotransferase,^8)^ pyrophosphate-dependent phosphofructokinase,^9)^ poly(P)-dependent riboflavin kinase,^10)^ pyrophosphate-forming acetate kinase,^11)^ and adenosine 5′-triphosphate (ATP)/poly(P)-dependent gluco(manno)kinase.^12,13)^ In this review, the earlier mentioned enzymes and ATP/poly(P)-NAD kinase,^14)^ which we found in *Achromobacter butyri* in 1980, will be discussed. The occurrence of these poly(P)-dependent enzymes implies that poly(P) functioned in primitive organisms like an ATP molecule does in existing organisms and all poly(P)-dependent enzymes were thought to have been lost through natural selection in molecular evolution.

However, recent studies have demonstrated that poly(P) is a phosphate storage molecule or energy source. It has several significant physiological functions in various organisms. Poly(P) has now been considered an essential biochemical from prebiotic times to the present and is deemed a research hotspot.^5,15,16)^ Nicotinamide adenine dinucleotide (NAD^+^), nicotinamide adenine dinucleotide phosphate (NADP^+^), and their reduced forms, *i.e.*, NADH and NADPH, are essential molecules with pivotal cellular oxidation/reduction reactions. ATP-specific NAD kinase (ATP-NAD kinase) is the sole enzyme that phosphorylates NAD^+^ to NADP^+^ and has been examined extensively.^17,18)^ However, the study on NAD kinase is restricted to its biochemistry, especially the enzymatic activity regulation, even after we found a novel NAD kinase in 1980 that uses both ATP and poly(P) [ATP/poly(P)-NAD kinase] for NAD^+^ phosphorylation. This was presumably due to the scarcity of the gene encoding NAD kinase in all living organisms.

In 2000, we have first identified and cloned the NAD kinase gene from *Mycobacterium tuberculosis* H37Rv.^19)^ Although the cloned gene was for ATP/poly(P)-NAD kinase, and not ATP-NAD kinase, this led to a broad range of physiological, structural, and evolutionary studies on NAD kinase worldwide. Based on the biochemical and genetic characteristics of NAD kinases, we identified the long-awaited human mitochondrial NAD kinase,^20)^ *i.e.*, bacterial-type ATP/poly(P)-NAD kinase. This caused a provocative impact on the area of mitochondrial biology and diseases.

Our pioneering research results on NAD kinase, *i.e.*, findings of ATP/poly(P)-NAD kinase, NAD kinase gene, and human mitochondrial poly(P)-dependent NAD kinase, were able to confirm the function of poly(P) as an ATP substitute. They paved the way for future studies of NAD kinase, poly(P), and mitochondria.

From a personal viewpoint, this review will discuss the recent development in the physiological and molecular biological studies of NAD kinase in various organisms, including the background behind the findings of novel NAD kinase and its gene.

## 1 Pioneering research results on NAD kinase

The two pioneering research results, *i.e.*, discovery of the ATP/poly(P)-NAD kinase in 1980 in Gram-positive bacteria^14)^ and identification of the gene in 2000,^19)^ have significantly enhanced the study on biochemistry, molecular biology, and evolutionary biology of NAD kinase in various organisms.

### 1.1 Discovery of ATP/poly(P)-NAD kinase.

Poly(P) is a linear or cyclic homopolymer of orthophosphate residues linked by energy-rich phosphoanhydride bonds.^21)^ Cyclic phosphate polymers are often referred to as metaphosphates. The enzyme poly(P) kinase is responsible for the synthesis of poly(P) from terminal phosphate of ATP [ATP + poly(P)_n_ → adenosine 5′-diphosphate (ADP) + poly(P)_n+1_].^6)^ NAD^+^, NADP^+^, and their reduced forms, NADH and NADPH, function as cofactors involved in cellular oxidative-reductive reactions.^22,23)^ NAD^+^ and NADH mainly function in catabolic reactions, whereas NADP^+^ and NADPH are known to be involved in anabolic reactions and defense against oxidative stress.^24)^ NAD^+^ and NADP^+^ also function as substrates in several reactions.^22,23,25–28)^ For example, NAD^+^ supplies adenosine 5′-diphosphate-ribose moiety for the enzymatic synthesis of poly(ADP-ribose).^29)^ The presence of both non-phosphorylated (NAD^+^ and NADH) and phosphorylated (NADP^+^ and NADPH) forms may be significant in making it possible for several kinds of redox reactions to co-exist in a cell and/or to control traffic of metabolic pathways.^30)^

**Figure 1.**
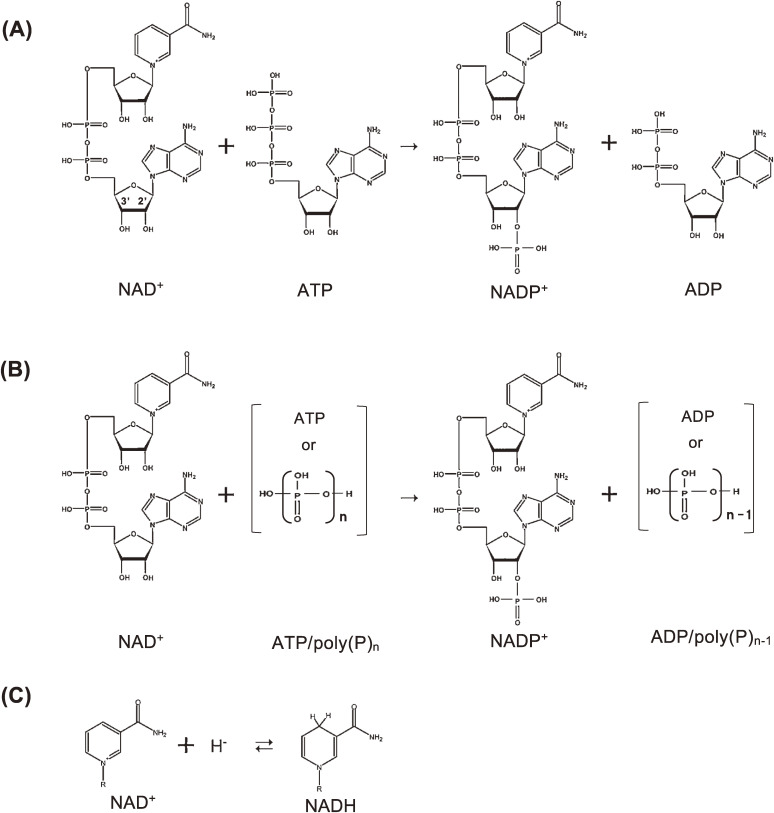
NAD kinase reactions. **(A)** ATP-NAD kinase reaction. NAD^+^ is phosphorylated to NADP^+^ by using only ATP. **(B)** ATP/poly(P)-NAD kinase reaction. NAD^+^ is phosphorylated to NADP^+^ by using ATP or poly(P). Linear and cyclic poly(P)s are then utilized. **(C)** Structures of NAD^+^ and NADH. A hydride ion (H^−^) is added to a nicotinamide moiety of NAD^+^, resulting in NADH. This figure was reproduced from S. Kawai and K. Murata (2008) Biosci. Biotechnol. Biochem. **72**, 919–930 (Ref. 63) with some modifications.

NAD kinase is the sole NADP^+^ biosynthetic enzyme and catalyzes the phosphorylation of NAD^+^ to NADP^+^ in the presence of ATP (ATP-NAD kinase: NAD^+^ + ATP → NADP^+^ + ADP) (Fig. 1A). The enzyme ATP-NAD kinase was discovered in 1950 by Arthur Kornberg, who partially purified it from the yeast *Saccharomyces cerevisiae*.^31)^ Subsequently, NAD kinases were purified from pigeon liver,^32)^ pigeon heart,^33)^ *S. cerevisiae*,^34)^ and yeast *Candida utilis*,^35)^ and some properties of the purified enzymes have been reviewed.^17,18)^ In addition, as for the specificity of the phosphoryl acceptor (NAD^+^ and NADH), NAD(H) kinase having higher affinity to NADH than to NAD^+^ has been known to reside in the mitochondria of a yeast *S. cerevisiae*^23)^ and in the peroxisome of a plant *Arabidopsis thaliana*.^36)^

In 1980, during the course of study on the production of NADP^+^ by using ATP-NAD kinase, we were able to find another NAD kinase that uses poly(P), especially metaphosphates, besides ATP, in cells of Gram-positive bacterium *Achromobacter butyri*^14)^ and named it ATP/poly(P)-NAD kinase [NAD^+^ + ATP/poly(P)_n_ → NADP^+^ + ADP/poly(P)_n−1_] (Fig. 1B). The enzyme was widely distributed among bacteria, especially in Gram-positive bacteria.^14)^ The distribution pattern of the ATP/poly(P)-NAD kinase was almost similar to that of ATP/poly(P)-glucokinase [glucose + ATP/poly(P)_n_ → glucose-6-phosphate (G-6-P) + ADP/poly(P)_n−1_],^14)^ thus suggesting that these bacteria have characteristic and pivotal systems for phosphate and energy metabolism. The ATP/poly(P)-NAD kinase, as well as ATP-NAD kinase, is known to specifically phosphorylate 2′-hydroxyl, but not 3′-hydroxyl, in ADP moiety of NAD^+^ molecule, yielding biologically active NADP^+^ (Figs. 1A and B).

The finding of ATP/poly(P)-NAD kinase undoubtedly gave a clue to expand the biochemical researches on NAD kinase, especially from the standpoint of molecular evolution of the enzyme. However, despite the physiological importance of NAD kinase and the fact that there has been accumulated substantial biochemical information on the ATP-NAD kinase, further biochemical, molecular biological, and evolutional studies of this enzyme have not made much progress, since the gene encoding NAD kinase had not been identified.

### 1.2 Identification of NAD kinase gene.

The ATP/poly(P)-NAD kinase was first isolated from a Gram-positive bacterium *Brevibacterium ammoniagenes*.^37)^ However, it remains undetermined whether the poly(P)- and ATP-dependent NAD kinase activities are catalyzed by the same enzyme or not. To make it clear, ATP/poly(P)-NAD kinase was purified from Gram-positive *Micrococcus flavus*, named Mfnk, and it was confirmed that both activities of Mfnk are exhibited by a single protein.^19)^

Furthermore, analysis of the N-terminal and internal amino acid sequences of the purified Mfnk indicated that the hypothetical gene Rv1695 of Gram-positive bacterium *M. tuberculosis* is responsible for ATP/poly(P)-NAD kinase, and the enzyme coded by Rv1695 gene was named Ppnk. A similar poly(P)-dependent NAD kinase gene, *Mfnk*, was also identified in *M. flavus*.^38)^ Ppnk of *M. tuberculosis* is the typical ATP/poly(P)-NAD kinase, of which structure and functions have been extensively examined by us^39–42)^ and by other researchers.^43,44)^

Thus, we, for the first time, identified the gene for NAD kinase in Gram-positive bacteria. Identifying the NAD kinase gene enabled us to search for its homolog in the genome sequences of many organisms such as Archaea, eubacteria, and eukaryotes, except for *Chlamydia trachomatis*, which has no gene homolog for pyridine metabolism.^45)^ Almost all organisms were found to contain only one NAD kinase gene. Alternatively, fission yeast *Schizosaccharomyces pombe* has four genes, whereas plant *A. thaliana*, budding yeast *S. cerevisiae*, and cyanobacterium *Synechococcus* sp. PCC6301 all have three genes. In contrast, opportunistic pathogenic yeast *Candida albicans*, protist *Dictyostelium discoideum*, and some eubacteria such as *Bacillus*, *Listeria*, *Streptomyces*, and other cyanobacteria contain two genes. Although humans have been thought to have one NAD kinase gene, we were able to determine that they contain two kinds of genes (see Section 4). The NAD kinases encoded by several homologs in one organism may be distributed to subcellular organelles, such as peroxisome, chloroplast, and mitochondria, to play pivotal and specific roles (see Section 3.2).

### 1.3 Molecular structure of NAD kinase.

NAD kinase genes (homologs) were cloned from several microbes,^19,38,42,46–53)^ plants,^36,54–56)^ and humans,^57,58)^ and their gene products (recombinant NAD kinases) were characterized (Table 1). This analysis resulted in the deduction of two crucial features. First, all NAD kinases take homomultimeric (homodimer, homo-tetramer, homo-hexamer, or homo-octamer) structures. The significance of the homomultimeric structures of NAD kinase was revealed by structural studies, which will be discussed in Section 3.1. Second, although the molecular size of the NAD kinase subunit is estimated to range from 30 (prokaryotic NAD kinase) to 40 kDa (eukaryotic NAD kinase), that of NAD kinase (MJ0917) from the archaeon *Methanocaldococcus jannaschii* (formerly *Methanococcus jannaschii*) is exceptionally large, which is approximately 64 kDa (Table 1).

This was caused by an overlap of two genes for NAD kinase and inositol monophosphatase (IMPase).^46,59–61)^ Thus, the IMPase gene product shows no IMPase activity, but exhibits NADPase activity and fructose-1,6-bisphosphatase activity. MJ0917 NAD kinase of *M. jannaschii* and MMP1489 NAD kinase of *M. maripaludis*^46)^ are bifunctional enzymes, NAD kinase/NADPase, with opposing dual activities, synthesis, and degradation of NADP^+^. This may be advantageous in regulating the intracellular concentration of NAD^+^ and NADP^+^, leading to an unprecedented finding of “intrinsic NADPase”. However, whether the fusion of the two proteins inevitably occurred remains unclear because the NAD kinase and IMPase are still occurring separately in the present genomic sequences of several euryarchaca.

Other enzymatic properties, especially phosphoryl donor and acceptor specificity of NAD kinase, have been described in Section 3.2 to facilitate the understanding of the relationship between the specificity and structure of their binding sites in NAD kinase.

## 2 Primary structure and evolution of NAD kinase

From the standpoint of poly(P) as an ancient energy and phosphorus carrier,^5,15,16)^ it is assumed that NAD kinase has evolved from ATP/poly(P)-NAD kinase to ATP-NAD kinase. This analysis of homology and sequence alignment of NAD kinases indicated a plausible evolution process of NAD kinases.^62)^

### 2.1 Primary structure of NAD kinase.

**Figure 2.**
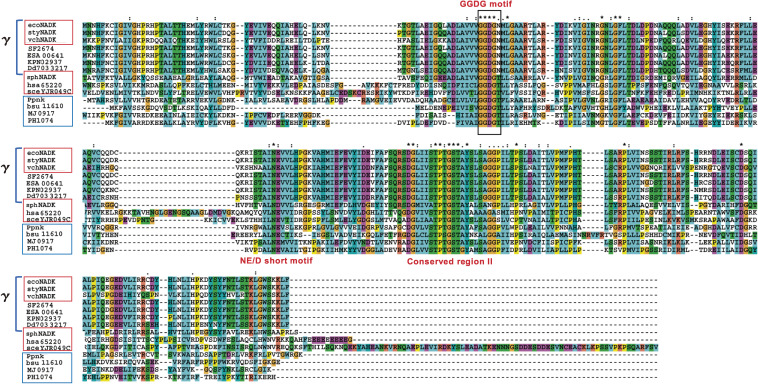
Multiple alignment of the primary structure of ATP-NAD kinases (boxed in red), ATP/poly(P)-NAD kinases (boxed in blue), and γ-proteobacterial NAD kinase homologs (indicated by γ).^62)^ ATP-NAD kinases are identified as follows: ecoNADK (*E. coli*), styNADK (*S. enterica*), vchNADK (*V. cholerae*), sphNADK (*Sphingomonas* sp. A1), hsa65220 (human), and sceYJR049C (*S. cerevisiae*). ATP/poly(P)-NAD kinases are Ppnk (*M. tuberculosis*), bsu11610 (*B. subtilis*), MJ0917 (*M. jannaschii*), and PH1074 (*P. horikoshii*). γ-Proteobacaterial NADK homologs are ecoNADK, styNADK, vchNADKs, SF2674 (*Shigella flexneri*), ESA00641 (*Cronobacter sakazakii*), KPN02937 (*Klebsiella pneumoniae*), and Dd703 3217 (*Dickeya dadantii*). The motifs GGDGN and GGDGT are boxed in black. The motif GGDGN is conserved in the primary structures of some γ-proteobacterial NAD kinase homologs, while the motif GGDGT is found in those of other ATP-NAD kinases and ATP/poly(P)-NAD kinases. Identical residues are denoted by an asterisk (*), strongly conserved residues by a colon (:), and weakly conserved residues by a period (.). This figure was reproduced from Y. Nakamichi *et al.* (2013) Sci. Rep. **3**, 2632 (Ref. 62, Supporting information) with some modifications.

**Figure 3.**
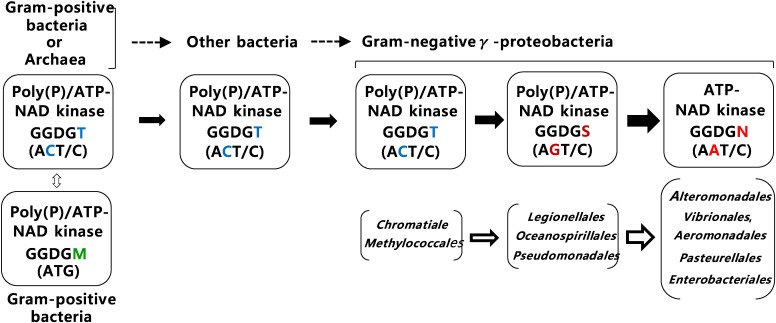
(Color online) Proposed evolution of bacterial NAD kinase from ATP/poly(P)-NAD kinase to ATP-NAD kinase.^62)^ The changes in GGDG motifs of NAD kinases from GGDGT via GGDGS into GGDGN and those in corresponding codons from ACT/C (Thr) via AGT/C (Ser) into AAT/C (Asn) during bacterial evolution are indicated by arrows, together with related Gram-positive and -negative bacteria. ATP/poly(P)-NAD kinase carrying the GGDGT motif, in which the Thr residue is encoded by ACT/C, but not by ACA/G, is assumed to be a direct ancestor of γ-proteobacterial ATP-NAD kinases carrying the GGDGN motif, in which Asn is encoded by AAT/C. ATP/poly(P)-NAD kinases carrying the GGDGM motif (in which the Met residue is encoded by ATG), which is concentrated within Gram-positive bacteria, is not a direct ancestor of γ-proteobacterial ATP-NAD kinases. This figure was reproduced from Y. Nakamichi *et al.* (2013) Sci. Rep. **3**, 2632 (Ref. 62, Supporting information) with some modifications.

ATP-NAD kinases are contained in Gram-negative α- and γ-proteobacteria and eukaryotes. On the other hand, ATP/poly(P)-NAD kinases are found in Archaea and Gram-positive bacteria (Figs. 2 and 3). However, the homology search for amino acid sequences (*BLASTP* analysis) has indicated that the primary structures of both NAD kinases, *i.e.*, ATP-NAD kinase and ATP/poly(P)-NAD kinase, are closely similar to each other.^62)^ For example, the primary structure of hsa65220, a human cytosolic ATP-NAD kinase (hsa means *h**omo* *sa**piens*) is similar to that of the ATP-NAD kinases of Gram-negative γ-proteobacteria [ecoNADK (YfjB) of *Escherichia coli*: 49% similarity over 259 residues, E = 3e-22; styNADK of *Salmonella enterica*: 49% over 259 residues, E = 9e-23; vchNADK of *Vibrio cholerae*: 48% over 330 residues, E = 1e-28] and to that of the ATP/poly(P)-NAD kinases of an archaeon (PH1074 of *Pyrococcus horikoshii*: 46% similarity over 277 residues, E = 4e-18) and a Gram-positive bacterium (Ppnk of *M. tuberculosis*: 49% similarity over 228 residues, E = 1e-18).^62)^ However, despite these similarities, it remains difficult to identify the key conserved amino acid residues that make the sharp distinction between ATP-NAD kinases and ATP/poly(P)-NAD kinases.

Three conserved motifs, that is, the GGDG motif, the NE/D short motif, and the conserved region II, were determined in the primary structures of all NAD kinases (Fig. 2). Among these, we focused on the highly conserved GGDG motifs,^38,62,63)^ because this motif is also conserved in diacylglycerol kinases and 6-phosphofructokinase and is thought to participate in catalysis of these kinases.^63,64)^ Interestingly, the GGDGN (GGDG plus N) motif is conserved in the primary structures of the γ-proteobacterial NAD kinases, such as ecoNADK, styNADK, and vchNADK. In these enzymes, asparagine (Asn) residues in the GGDGN motif are Asn-75 and Asn-76, respectively. Furthermore, the GGDGN motif is determined in 292 NAD kinases, among which 291 are restricted to the most recently diverged γ-proteobacteria,^65)^ whereas the remaining one is from an ε-proteobacterium (*Campylobacter fetus*). In contrast, the GGDGT (GGDG plus T) motif has been identified to be more prevalent in ATP- and ATP/poly(P)-NAD kinases. The motif was found in 254 of 286 eukaryotic NAD kinases, 1,100 of 1,836 bacterial NAD kinases, and 124 of 127 archaeal NAD kinases.

The GGDGN motif is observed in the most recently diverged γ-proteobacterial NAD kinase, probably ATP-NAD kinase, strongly suggesting that the ATP-NAD kinase has evolved from ATP/poly(P)-NAD kinase. To shed light into this evolutionary process, the role of Asn residue in GGDGN motif was analyzed by converting the Asn residue of ecoNADK (Asn-75) and vchNADK (Asn-76) to threonine (Thr) residues.

### 2.2 Evolution of NAD kinase.

Taking into consideration that biological systems may have used the energy-rich phosphates in poly(P) before the evolution of ATP,^21)^ it is reasonable to assume that NAD kinase has evolved from ATP/poly(P)-NAD kinase into ATP-NAD kinase, and recently diverged organisms obtained ATP-NAD kinase during their evolution. The deduced evolutionary process of microbes [described in (i) as follows] and the facts shown in Sections 1 and 2.2 regarding the distribution pattern of ATP/poly(P)-NAD kinase and ATP-NAD kinase [summarized in (ii) as follows] will support this assumption: (i) Bacteria evolved from Gram-positive bacteria or Archaea into Gram-negative γ-proteobacteria in the following order: Gram-positive bacteria (low G + C content) (↔Archaea) → Gram-positive bacteria (high G + C) → others (Gram-negative bacteria except for proteobacteria) → Gram-negative ε- and δ-proteobacteria → Gram-negative α-proteobacteria → Gram-negative β-proteobacteria → Gram-negative γ-proteobacteria.^65,66)^ (ii) ATP/poly(P)-NAD kinases are distributed throughout Gram-positive bacteria (*e.g.*, *M. tuberculosis* and *Bacillus subtilis*) and Archaea (*e.g.*, *M. jannaschii* and *P. horikoshii*). Alternatively, ATP-NAD kinases are retained in Gram-negative α-proteobacterium (*Sphingomonas* sp. A1),^48)^ Gram-negative γ-proteobacteria (*E. coli* and *S. enterica*), and eukaryotes including fungi, plants, and humans (Fig. 2).^19,36,45–52,56,57)^

How did NAD kinases evolve from ATP/poly(P)-NAD kinases into ATP-NAD kinases? There is no doubt that the presence or absence of poly(P) utilization ability holds the key to understanding NAD kinase evolutionary mechanism. Thus, our interest was directed toward the function of Asn residue in the GGDGN motif in ATP-NAD kinase of Gram-negative γ-proteobacteria (*E. coli* and *V. cholerae*), the most recently diverged group as indicated in Section 2.1, and that of Thr residue in the GGDGT motif in ATP/poly(P)-NAD kinases of Gram-positive bacteria (*M. tuberculosis* and *B. subtilis*) and Archaea (*M. jannaschii* and *P. horikoshii*) (Fig. 2).

NAD kinases with the Asn residue in the GGDGN motif are often restricted to γ-proteobacteria (Fig. 2). Furthermore, the Gram-negative γ-proteobacteria have evolved in the following order: *Chromatiales*, *Methylococcales*, *Xanthomonadales*, *Thiotrichales* → *Oceanospirillales*, *Pseudomonadales* → *Alteromonadales* → *Vibrionales*, *Aeromonadales* → *Pasteurellales* → *Enterobacteriales*.^67)^ Almost all γ-proteobacterial NAD kinases carrying the GGDGN motif are noted in the most recently diverged group within γ-proteobacteria (*Alteromonadales*, *Vibrionales*, *Aeromonadales*, *Pasteurellales*, and *Enterobacteriales*), thus decisively indicating that the NAD kinases with the GGDGN motif are in the forefront of NAD kinase evolution (Fig. 3).

On the other hand, the evolution of NAD kinase with the Thr residue in GGDGT motif has been found to be more complicated than that of NAD kinase (*i.e.*, ATP-NAD kinase) with the GGDGN motif. ATP/poly(P)-NAD kinases contain, for example, a GGDGS or GGDGM motif, in addition to a GGDGT motif. However, it seems likely that this diversity of motifs is exactly a driving force for NAD kinase evolution following the evolutionary process of microbes (Fig. 3).

NAD kinases containing the GGDGT motif in Archaea (NAD kinases PH1074 of *P. horikoshii* and MJ0917 of *M. jannaschii*), Gram-positive bacteria [NAD kinases bsu11610 (a.k.a., NadF) of *B. subtilis* and Ppnk of *M. tuberculosis*], and ecoNADK N75T of *E. coli* are all ATP/poly(P)-NAD kinases.^19,47,51,52)^ This indicates that all prokaryotic NAD kinases carrying the GGDGT motif, except for some α-proteobacterial NAD kinases, are ATP/poly(P)-NAD kinases. On the other hand, the 124 NAD kinases containing the GGDGS motif exist over Gram-positive bacteria (approximately 20%: *i.e.*, 24 enzymes) and γ-proteobacteria (about 80%: *i.e.*, 79 enzymes), but are not found in other proteobacteria. Moreover, the 80 NAD kinases carrying the GGDGM motif are almost entirely restricted to Gram-positive bacteria (about 100%: *i.e.*, 79 enzymes). Furthermore, ecoNADK N75S and ecoNADK N75M show ATP/poly(P)-NAD kinase activity, suggesting that the bacterial NAD kinases with the GGDGS or GGDGM motif are also ATP/poly(P)-NAD kinases. The aforementioned observations indicate that almost all NAD kinases of Archaea and bacteria that would probably be branched at the deeper point than γ-proteobacteria^65–67)^ are determined to be ATP/poly(P)-NAD kinases.

On the basis of the aforementioned experimental results and considerations, the evolution process of NAD kinase could be postulated as follows: ATP/poly(P)-NAD kinase (with GGDGT motif) → Gram-negative γ-proteobacterial ATP/poly(P)-NAD kinase (with GGDGS motif) → Gram-negative γ-proteobacterial ATP-NAD kinases (with GGDGN motif). The change in amino acid residues corresponds to the evolution of codons reaching the GGDGN motif, in which the Asn residue is coded by AAT/C as follows: ACT/C (Thr) → AGT/C (Ser) → AAT/C (Asn). This evolution process of NAD kinases indicates that ATP/poly(P)-NAD kinase carries the GGDGT motif. Furthermore, the Thr residue encoded by ACT/C, but not by ACA/G, seems to be the direct ancestor of γ-proteobacterial ATP-NAD kinases. Likewise, ATP/poly(P)-NAD kinase carrying the GGDGM motif, in which the methionine (Met) residue is encoded by ATG, is not the direct ancestor.^62)^

In addition to the GGDGT motif, α-proteobacterial NAD kinases are known to contain various motifs such as GGDGF, GGDGL, and GGDGE. This enables α-proteobacterial NAD kinases to evolve in a different route than γ-proteobacterial NAD kinases. This notion is typically seen in the following facts: the NAD kinase sphNADK (a.k.a., NadK) of α-proteobacterial *Sphingomonas* sp. A1 contains the GGDGT motif but is an ATP-NAD kinase.^48)^ The NAD kinase C5orf33 of human mitochondria, which is believed to have originated from α-proteobacteria,^68)^ carries the GGDGT motif, but is an ATP/poly(P)-NAD kinase,^20)^ as described in Section 4. In contrast, NAD kinases of eukaryotes (*e.g.*, humans and plants), except for the human mitochondrial ATP/poly(P)-NAD kinase C5orf33 noted earlier,^20)^ act as ATP-NAD kinases. However, they contain the GGDGT motif.^36,49,50,56,57)^ Thus the eukaryotic NAD kinases may have evolved into ATP-NAD kinases from the ATP/poly(P)-NAD kinase through a more complicated process.

Thus, in this study, we demonstrated the molecular routes through which bacterial NAD kinases have evolved from ATP/poly(P)-NAD kinases to γ-proteobacterial ATP-NAD kinases. A more detailed analysis of phylogenetic trees of proteobacteria, NAD kinase evolutionary velocity, and crystal structures of NAD kinases would further contribute to the elucidation of this evolutionary mechanism.

## 3 Tertiary structure and determinant of substrate specificity of NAD kinase

NAD kinase genes (homologs) were cloned from microorganisms,^19,24,38,42,46,48,49,51–53)^ plants,^54–56)^ and humans.^57)^ Their homomultimeric structures (homodimer, homo-tetramer, homo-hexamer, or homo-octamer) were mainly determined using recombinant enzymes (Table 1). In addition to these common structural properties, NAD kinase are known to exhibit different specificities, depending on the phosphoryl donors and acceptors of ATP-NAD and ATP/poly(P)-NAD kinases (Table 1), which are as follows [(i)–(iii)]: (i) NAD kinases from Gram-positive bacteria and Archaea use ATP and poly(P) and phosphorylate NAD^+^ and NADH. (ii) NAD kinases from eukaryotes use ATP, but not poly(P), and phosphorylate NAD^+^ and NADH. (iii) NAD kinases from Gram-negative bacteria use ATP, but not poly(P), and phosphorylate NAD^+^, but not NADH.

The necessity of a homomultimeric structure for activity expression and the structural determinants for the phosphoryl donor (ATP or poly(P)) and acceptor (NAD^+^ and NADH) specificity are discussed in this section, which is mainly based on the tertiary structure of ATP/poly(P)-NAD kinase.

### 3.1 Tertiary structure of ATP/poly(P)-NAD kinase.

Why do all NAD kinases have to take a homomultimeric structure? Our identification of the NAD kinase gene in 2000^19)^ has caused intense competition to solve this question in structural biology (X-ray crystal structural analysis). We determined the tertiary structures of the apo and holo (complex with NAD^+^) forms of ATP/poly(P)-NAD kinase (Ppnk) from *M. tuberculosis*.^39–42)^ Apo form of Ppnk was also determined by Garavaglia *et al.*^43)^ Likewise, other researchers have clarified the tertiary structures of the holo forms of ATP/poly(P)-NAD kinase (Afnk) from the hyperthermophilic archaeon *Archaeoglobus fulgidus*,^69)^ the holo forms of ATP-NAD kinase (LmNADK1) from *Listeria monocytogenes* (eubacterium),^70)^ and an apo form of ATP/poly(P)-NAD kinase from the hyperthermophilic eubacterium *Thermotoga maritima*.^71)^

**Figure 4.**
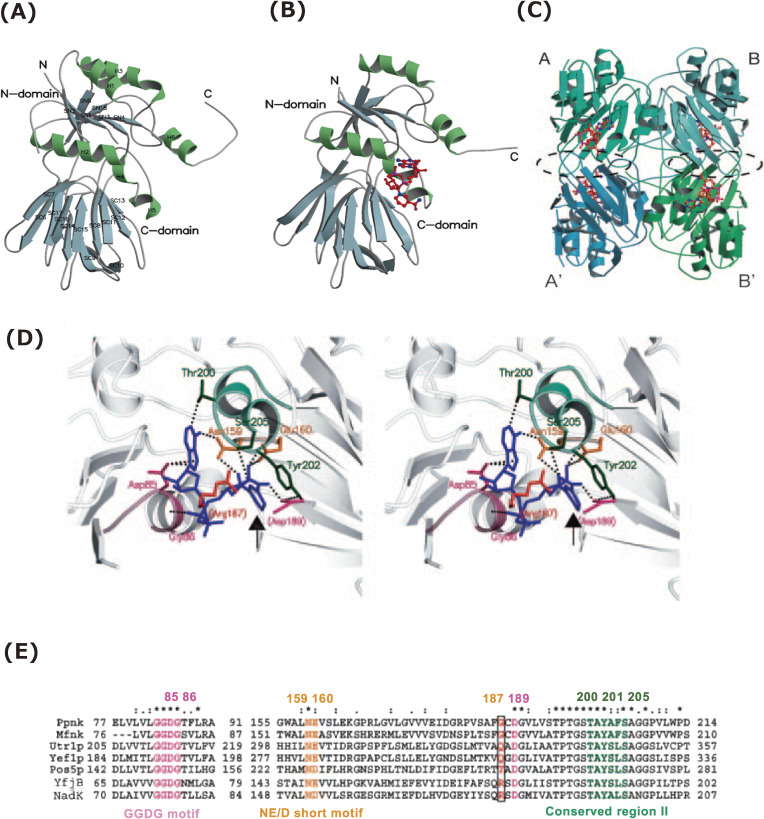
Structure of NAD kinase and structural determinant of phosphoryl acceptor specificity.^39–41,63)^ **(A)** A ribbon model of apo-Ppnk. The α-helix (H) and β-strand (SN and SC for N- and C-domains) are designated by numbers from N-terminal. **(B)** A ribbon model of holo-Ppnk (Ppnk-NAD). NAD is indicated in red (nitrogen, blue; phosphorus, pink). **(C)** A ribbon model of the quaternary structure of Ppnk-NAD. An asymmetric unit contains two subunits (Ppnk-NAD-A/-B or -A′/-B′). NAD^+^ is shown to be similar to (B). The A-A′ and B-B′ intersubunit contact regions are enclosed by a broken black line. **(D)** Stereodiagram of the binding site for NAD^+^ (blue) in Ppnk with the exception of Arg-187. Arg-187 is artificially replaced with Gly-187 in Ppnk. Asp-189 and Arg-187 are provided by the adjacent subunit and are denoted in parentheses. Residues of Gly-83 to Gly-86 (GGDG motif), Asn-159 to Glu-160 (NE/D short motif), and Thr-200 to Ser-205 (in conserved region II) are indicated in purple, yellow, and green, respectively. Contacts are represented by dotted lines. The nicotinamide ring of NAD^+^ is emphasized using an arrow. **(E)** Multiple alignment of the primary structures of NAD(H) kinases (Ppnk, Mfnk, Utr1p, Yef1p, and Pos5p) and NAD kinases [YfjB (ecoNADK) and NadK (sphNADK)]. The primary structures corresponding to the NAD^+^-binding site of Ppnk are aligned using ClustalW. The number of residues for each enzyme is specified. The residues participating in NAD^+^ binding are colored as in (C), while the important residues corresponding to Gly-187 in Ppnk are boxed. Identical residues are denoted by an asterisk (*), strongly conserved residues by a colon (:), and weakly conserved residues by a period (.). This figure was reproduced from S. Kawai and K. Murata (2008) Biosci. Biotechnol. Biochem. **72**, 919–930 (Ref. 63) with some modifications.

To uncover the correlations between the structure and function of NAD kinase, we focused on the tertiary structure of *M. tuberculosis* ATP/poly(P)-NAD kinase (Ppnk), which consists of 307 amino acid residues and takes a homotetrameric quaternary structure^19)^ (Fig. 4C). The apo form of Ppnk (apo-Ppnk) (Fig. 4A) and the holo form of Ppnk complexed with NAD^+^ (Ppnk-NAD) (Fig. 4B) consisted of two domains (N-domain: residues 1–138 and 279–283 and C-domain: residues 139–278) and a C-terminal tail (residues 284–307).^41)^ In the NAD^+^-binding site in Ppnk-NAD, the nicotinamide ring is stacked by tyrosine (Tyr)-202 at a distance of 3.3 Å and through a π-π interaction (Figs. 4B and C). Furthermore, aspartic acid (Asp)-189 from the adjacent subunit has been found to contribute to the correct orientation of the Tyr-202 and interact with an amide group in the nicotinamide moiety in subunit A in Ppnk. These indicate that the A-A′ or B-B′ intersubunit contact, *i.e.*, the formation of A-A′ or B-B′ homodimer structure, is essential in creating the NAD^+^-binding site in Ppnk (Fig. 4C).

In the primary structures of Ppnk, the amino acid residues necessary for the formation of NAD^+^-binding site are conserved; they create three conserved motifs, viz., the GGDG motif [glycine (Gly)-83 to Gly-86], the NE/D short motif [Asn-159 to glutamic acid (Glu)-160], and the conserved region II [Asp-189 to valine (Val)-210] (Figs. 2 and 4E). The site-directed mutagenesis indicated that amino acid residues, Asp-85 (in GGDG motif), Asn-159 (in NE/D short motif), and Glu-160, Thr-200, and Tyr-202 (in conserved region II) are vital in the activity expression of Ppnk.^41,44)^ In ATP-NAD kinase (LmNADK1) from *L. monocytogenes*, it was shown that Asp-45 in the GGDG motif subtracts a proton from 2′-hydroxyl, but not 3′-hydroxyl, of NAD^+^ to activate the phosphate acceptor.^70)^ Therefore, Asp-85 in the GGDG motif of Ppnk should have the same function as Asp-45 in the GGDG motif of LmNADK1. Furthermore, the occurrence of substrate-assisted catalysis, in which the diphosphate group in NAD^+^ plays a significant role in catalysis,^70)^ was also emphasized in the LmNADK1 reaction.

The necessity of the formation of A-A′ or B-B′ homodimer structure in NAD kinase (Fig. 4C) has also been recognized in the case of ATP-NAD kinase (Afnk) from archaeon *A. fulgidus*. Briefly, in Afnk, the AMP portion of ATP binds to the same binding site as the nicotinamide ribose moiety of NAD^+^ and NADP^+60^ and Asp-145, which corresponds to Asp-189 of Ppnk and Asp-150 of LmNADK1, from the adjacent subunit interacts with the adenine ring of ATP and nicotinamide rings of NAD^+^ and NADP^+^. These further indicate that the intersubunit contact, *i.e.*, the formation of homodimer structure, is indispensable for creating NAD^+^- and ATP-binding sites in NAD kinases.

### 3.2 Phosphoryl donor and acceptor specificity.

**Figure 5.**
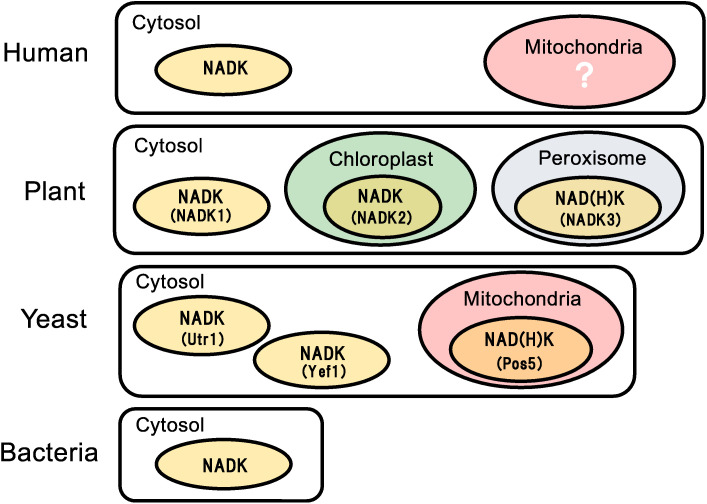
(Color online) NAD kinase in human, plant, yeast, and bacteria. Localization of NAD kinase (abbreviated to NADK in this figure) in human (*Homo sapiens*), plant (*A. thaliana*), yeast (*S. cerevisiae*), and bacteria is illustrated. Human is found to contain only one NAD kinase in cytosol. Plant *A. thaliana* has three kinds of NAD kinases, NADK1 in cytosol, NADK2 in chloroplast, and NADK3 in peroxisome. Yeast *S. cerevisiae* has three kinds of NAD kinases, Utr1 and Yef1 in cytosol and Pos5 in mitochondria. NADK3 and Pos5 show high affinity to NADH (*i.e.*, NAD(H) kinase indicated as NAD(H)K in this figure). Bacteria have only one NAD kinase in cytosol. Note that NAD kinase in the mitochondria of the human cell is marked with a question sign, because the identification of the kinase is a direct theme of this study. Specifically, it signifies C5orf33 (*i.e.*, ATP/poly(P)-NAD(H) kinase), as explained in Section 4.

Poly(P) has been considered to function as an ATP substitute, and the properties of poly(P)-related enzymes have been well documented in some organisms.^15,72)^ The phosphoryl donor and acceptor specificity of ATP/poly(P)-NAD kinase and ATP-NAD kinase are thought to be differentially regulated following the cellular redox levels, localization sites, or both, such as the cytosol and organelles (Fig. 5).

#### Phosphoryl donor.

ATP-NAD kinases, such as ecoNADK (YfjB) of *E. coli* and hsa65220 in humans (cytosol), specifically phosphorylate NAD^+^ to NADP^+^ using ATP.^47,48)^ However, NAD kinases, *i.e.*, Mfnk of *M. flavus*,^19)^ Ppnk of *M. tuberculosis*,^19)^ and NAD kinase C5orf33 of human mitochondria^20)^ (described in Section 4) are ATP/poly(P)-NAD kinase that are known to phosphorylate NAD^+^ to NADP^+^ using poly(P) with ATP (Table 1).

Among the commercially available poly(P)s, cyclic poly(P)s, such as trimetaphosphate, tetrametaphosphate, hexametaphosphate, and metaphosphate (mixtures of various cyclic poly(P)s) are efficient phosphoryl donors for ATP/poly(P)-NAD kinases than linear poly(P)s. However, specifically determining the activity toward poly(P)s has remained to be a challenge, because poly(P)_n−1_ formed after the reaction can serve as a new phosphoryl donor substrate. The Ppnk of *M. tuberculosis* uses (activity against ATP: 100%) tetrapolyphosphate (163), hexametaphosphate (118), metaphosphate (178), and polyphosphate (mixtures of various linear poly(P)s) (151). Human mitochondrial NAD kinase (C5orf33, as discussed in Section 4) uses (ATP: 100%) tetrapolyphosphate (84), hexametaphosphate (194), and metaphosphate (280). On the other hand, ATP/poly(P)-NAD kinases of *A. butyri*^14)^ and *B. ammoniagenes*^37)^ specifically use cyclic poly(P)s, but not linear ones, as phosphoryl donors.

NAD kinase uses nucleoside 5′-triphosphates as phosphoryl donors for phosphorylating NAD^+^. For example, Ppnk of *M. tuberculosis* uses the following (ATP: 100%): deoxyadenosine triphosphate (dATP) (91), guanosine triphosphate (GTP) (88), cytidine triphosphate (CTP) (73), and uridine triphosphate (UTP) (87). NAD kinase isolated from some coryneform bacteria can use G-6-P as a phosphoryl donor.^73)^ However, this activity was not found in the cases of ATP/poly(P)-NAD kinases, Mfnk of *M. flavus*, and Ppnk of *M. tuberculosis*. Moreover, *p*-nitrophenyl phosphate was not used by ATP-NAD and ATP/poly(P)-NAD kinases, indicating that NAD^+^ phosphorylation could not be attributed to the reverse (phosphatase) reaction of the enzyme.^74)^ In the absence of NAD^+^, both ATP/poly(P)-NAD kinases, Mfnk and Ppnk, showed little phosphatase activity against ATP and tetrapolyphosphate.^19)^ The phosphoryl donor specificity of ATP/poly(P)-NAD kinase was almost comparable with that of poly(P)/ATP-glucokinases.^75,76)^

#### Phosphoryl acceptor.

NAD kinase from Gram-negative bacteria specifically phosphorylates NAD^+^, whereas NAD kinase from other organisms phosphorylates NAD^+^ and NADH (Table 1). The absence of NADH-phosphorylating activity (*i.e.*, NADH kinase activity) in NAD kinases [ecoNADK (YfjB) of *E. coli* and styNADK of *S. enterica*] from the Gram-negative bacteria agrees with the fact that NADH and NADPH are potent inhibitors of ecoNADK and styNADK.^45,47)^ In brief, NADH is a regulator of NAD kinase in these Gram-negative bacteria, but not a substrate.

However, NAD kinases from Gram-positive bacteria (Mfnk of *M. flavus* and Ppnk of *M. tuberculosis*) and eukaryotes (Utr1p and Yef1p of yeast *S. cerevisiae*) are found to be not regulated by NADH^19,49,50)^ and show NADH kinase activities. Notably, the NAD kinases Pos5 in yeast mitochondria,^24,53)^ NADK3 in plant peroxisome,^36)^ and human mitochondrial NAD kinase (C5orf33, as discussed in Section 4) show significantly higher NADH-phosphorylating activity than those in the cytosol or other organelles (Table 1 and Fig. 5). No evident phosphorylation of adenosine, AMP, ADP, and ADP-ribose by ATP/poly(P)-NAD kinase was observed.

### 3.3 Structural determinants of phosphoryl acceptor specificity.

As described in Section 3.2, ATP-NAD kinase from Gram-negative bacteria only phosphorylates NAD^+^, whereas ATP/poly(P)-NAD kinase from other organisms phosphorylates both NAD^+^ and NADH. The mechanism underlying the preferential use of NADH as a phosphoryl acceptor is found in the tertiary structure of Ppnk, an ATP/poly(P)-NAD kinase. To facilitate the explanation, the NAD kinase that phosphorylates NAD^+^ and NADH is designated as NAD(H) kinase.

In Ppnk (*i.e.*, NAD(H) kinase), the amino acid residue Gly-187 is identified as a structural determinant of phosphoryl acceptor specificity between NAD kinase and NAD(H) kinase^41)^ (Fig. 4D). The Gly-187 from the adjacent subunit was located around the nicotinamide ring in the tertiary structure of Ppnk-NAD, and it differed in terms of the primary structures of ATP-NAD kinases [arginine (Arg)-175 in ecoNADK of *E. coli* and Arg-180 in sphNADK (NadK) of *Sphingomonas* sp. A1^48)^]. In other ATP/poly(P)-NAD kinases (*i.e.*, NAD(H) kinase), the Gly-187 in Ppnk was determined to be replaced with Gly or polar amino acids: Gly-183 in Mfnk, glutamine (Gln)-330 in Utr1p, Gln-309 in Yef1p, and Thr-254 in Pos5p (Fig. 2).

Considering that NAD^+^ and NADH differ only in the nicotinamide moiety (Fig. 1C), it is expected that ATP-NAD kinase could be converted into NAD(H)-kinase through a single amino acid substitution, which corresponds to Gly-187 in Ppnk. ecoNADK R175G, in which Arg-175 was converted to Gly-175; ecoNADK R175Q (Q: glutamine); ecoNADK R175T; and ecoNADK R175H (H: histidine) were found to exhibit NAD kinase and NAD(H) kinase activities. Similarly, sphNADK R180G demonstrated NAD kinase and NAD(H) kinase activities. In contrast, ecoNADK R175K (K: lysine), ecoNADK R175E (E: glutamic acid), and ecoNADK R175I (I: isoleucine) showed no NAD(H) kinase activity. Thus, the strict substrate specificity of the ATP-NAD kinase was relaxed through a single amino acid substitution, that is, by converting NAD kinase to NAD(H) kinase.

Conversely, it was expected that NAD(H) kinase is converted to NAD kinase. However, Mfnk G183R, in which Gly-183 in Mfnk corresponds to Arg-175 in ecoNADK, still retained NAD(H) kinase activity. However, both activities to phosphorylate NAD^+^ and NADH were noted to decrease to 10% and 18%, respectively, compared with those of the native Mfnk.^41)^ Although NAD(H) kinase was not converted to NAD kinase, this result indicated that Gly-183 is not the sole determinant of the substrate (phosphoryl acceptor) specificity of Mfnk.

Collectively, these results obtained from the functional analysis of the Gly-187 in Ppnk confirmed that the residue from the adjacent subunit is a determinant of phosphoryl acceptor specificity. Also, intersubunit contact, *i.e.*, the formation of the homodimer structure, is significant in creating NAD^+^ and ATP-binding sites and in determining phosphoryl acceptor specificity in NAD and NAD(H) kinases. This study clearly explains the significance of homomultimeric structures of NAD kinase and NAD(H) kinases.

Thus, our pioneering findings of ATP/poly(P)-NAD kinase in 1980^14)^ and its gene in 2000^19)^ provided an essential clue to significantly enhance the biochemistry, molecular biology, and evolutionary biology of NAD kinase. The identification of NAD kinase gene has significantly paved the way for NAD kinase study. However, it raised some intriguing but exciting questions as follows: Why do bacteria maintain the ATP/poly(P)-NAD kinase? Why do the ATP/poly(P)-NAD kinase in microbes fail to evolve to ATP-NAD kinase? What are the ATP/poly(P)-NAD kinase functions in cells? Why NAD kinase is not detected in the human mitochondria, despite the study by many researchers (Fig. 5)? Human mitochondria are believed to have originated from α-proteobacteria through intracellular symbiosis,^77)^ especially with Gram-negative bacteria belonging to Rickettsiaceae having TCA (tricarboxylic acid) cycle, but not glycolysis.^78)^

## 4 Discovery of long-awaited human mitochondrial NAD kinase

Carrying out cellular respiration has been identified as the main function of mitochondria in eukaryotic cells. In the presence of oxygen, they turn nutrients supplied from the cells into carbon dioxide and water with a generation of energy (ATP). During this respiration process, however, reactive oxygen species (ROS) such as superoxide anion radical, hydroxyl radical, hydrogen peroxide, and singlet oxygen are produced, allowing the cells to develop various systems to eliminate these toxic species, especially by using pyridine nucleotides as universal electron carriers in mitochondrial electron transfer reactions. Although several lines of evidence have indicated that NAD kinase is involved in the elimination of mitochondrial ROS, for some reason, NAD kinase has remained unidentified in human mitochondria (Fig. 5).

Prokaryotes contain one NAD kinase in cytosol (Fig. 5). Yeast *S. cerevisiae* cells have three NAD kinases: two (Utr1 and Yef1) in the cytosol and one (Pos5) in the mitochondria^79,80)^ (Fig. 5). The NAD kinase triple mutant (pos5 utr1 yef1) is deemed lethal,^79)^ emphasizing the significance of intracellular NADP(H) biosynthesis. The phenotypes of the Pos5 mutant (pos5), which are probably caused by decreased level of mitochondrial NADPH, indicate that Pos5 is responsible for mitochondrial NADP(H) synthesis.^81)^ Plant *A. thaliana* has three NAD kinases, that is, NADK1 in the cytosol, NADK2 in the chloroplast, and NADK3 in the peroxisome^82)^ (Fig. 5). Furthermore, Pos5 in yeast mitochondria^24)^ and NADK3 in plant peroxisome^36)^ are classified as NAD(H) kinases having higher affinity to NADH than to NAD^+^, thus indicating the significance of NADPH supply for eliminating ROS in mitochondria and peroxisome.

**Figure 6.**
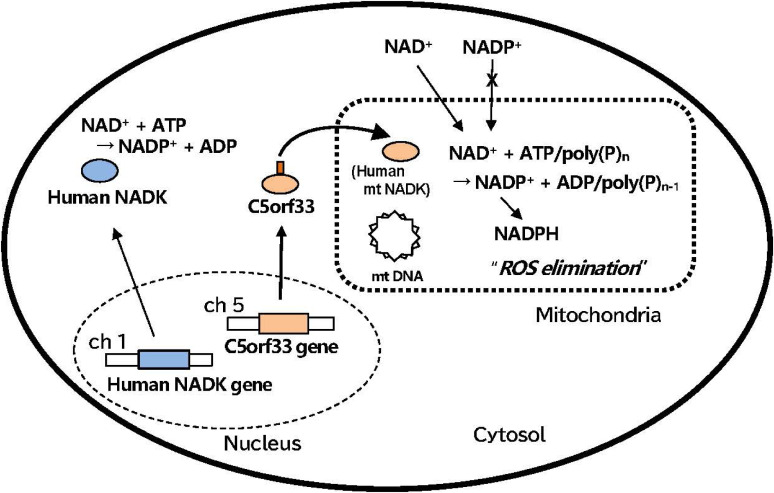
(Color online) Biosynthesis and localization of poly(P)-dependent human mitochondrial NAD kinase.^20)^ NAD^+^ is incorporated into mitochondria through the human mitochondrial membrane, but NADP^+^ is not. Human NAD kinase (blue circle) and mitochondrial NAD kinase (orange circle) are encoded by human NAD kinase gene on chromosome 1 (ch 1) (blue rectangle) and C5orf33 gene on chromosome 5 (ch 5) (orange rectangle) in human genome, respectively. The exon/intron structure and splicing process of the two genes are not included in this figure. The product C5orf33 (orange circle) of C5orf33 gene is delivered to mitochondria using a mitochondrial transit sequence (brown stick) and functions as poly(P)-dependent mitochondrial NAD kinase. Reactions catalyzed by human NAD kinase and human mitochondrial NAD kinase are given in cytosol and mitochondria, respectively. NADPH formed from NADP^+^ is utilized for elimination of reactive oxygen species (ROS). mt DNA, mitochondrial circular DNA; dotted square, mitochondria; dotted circle, nucleus. See text for further details.

In contrast to the cases in yeasts and plants, only a single gene encoding NAD kinase has been identified in the human genome. The product of this gene, *i.e.*, a human NAD kinase, is localized to the cytosol^58)^ (Figs. 5 and 6). NAD^+^ is incorporated into mitochondria through the human and mammalian mitochondrial membrane, but NADP^+^ is not^83)^ (Fig. 6). In fact, a mammalian transporter SLC25A51 (also known as MCART1) capable of importing intact NAD^+^ into mitochondria has been identified recently.^84)^ If these findings are accurate, human cells are at the level of prokaryotes with respect to the NAD kinase (Fig. 5). However, extensive studies searching for NAD kinase in human mitochondria have been conducted by many researchers but with no success, and the source of human mitochondrial NADP^+^ has remained elusive despite its great significance. Under these circumstances, in 2007, Pollak *et al.*^22)^ stated that “*given the vital role of at least two NAD kinase isoforms in yeast*, *one would certainly expect more than one mammalian NAD kinase isoform; furthermore*, *any additional NAD kinase isoforms in mammals should have primary structures substantially different from virtually all previously identified enzymes*, *at least according to the currently available information*”.

In parallel with the study based on the biochemistry and structural biology of microbial NAD kinases, we performed a detailed phylogenetic analysis of NAD kinase homologs, including earlier mentioned characteristics on primary and tertiary structures of bacteria, yeast, plant, and their evolution processes (Sections 2 and 3) and noted that, although the structural features of NADK3 homologs have not been reported to date, the primary structure and amino acids in the active center in plant NADK3 homologs, including *A. thaliana* NADK3, significantly differ from those of other typical NAD kinases, including mitochondrial Pos5 and cytosolic Utr1^50)^ in yeast. Thereafter, the molecular phylogenetic analysis based on the homology search using the primary structure of NADK3 of *A. thaliana* as a query was performed, and it was indicated in 2012 that C5orf33 protein is a novel human mitochondrial NAD kinase that is responsible for the missing source of mitochondrial NADP^+^ in human cells.^20)^

As mentioned in the previous sections, the C5orf33 protein was confirmed to localize in the human mitochondria (Fig. 6); further, it is known to contain a GGDG motif, *i.e.*, GGDGT specifying ATP/poly(P)-dependent NAD kinase,^41,62)^ and it is then synthesized in the cytosol and delivered to mitochondria using a mitochondrial transit sequence (Fig. 6). The identified mitochondrial NAD kinase (*i.e.*, C5orf33 protein) strikingly differed from cytosolic NAD kinase, which is a tetramer and specific to ATP (Table 1). The mitochondrial NAD kinase is a dimer and uses poly(P) as a phosphoryl donor in addition to ATP. Furthermore, although the structure of NAD^+^-binding site^85)^ is not sufficiently clear, the affinity of mitochondrial NAD kinase to NAD^+^ is deemed extremely low (*K*_m_: ∼22 µM) compared with that of cytosolic NAD kinase, the *K*m of which is approximately 1.1 mM. While the C5orf33 knockdown has no effects on mitochondrial morphology and cell viability, but results in the increases in ROS,^20)^ this clearly indicates that mitochondrial NAD kinase is involved in the elimination of mitochondrial ROS. To the best of our knowledge, we, for the first time, found a human mitochondrial NAD kinase.

This provocative discovery, however, raises some questions that need to be addressed. Firstly, does C5orf33 NAD kinase have a NAD(H) kinase activity, as yeast mitochondrial Pos5 and plant peroxisome NADK3 do? Protein sequence alignment shows that C5orf33 NAD kinase is closely related to *A. thaliana* NADK3, which implies the association of NAD(H) kinase activity in C5orf33 NAD kinase. Although being preliminary, we confirmed that the C5orf33 NAD kinase has NAD(H) kinase activity. Secondly, is C5orf33 NAD kinase activity regulated by Ca^2+^/calmodulin? Although the human NAD kinase is reported to be a Ca^2+^/calmodulin-dependent and its activity enhanced in the presence of the effectors,^86)^ the human NAD kinase has been proven to be non-calmodulin-dependent. Therefore, it is important to confirm the effect of Ca^2+^/calmodulin on C5orf33 NAD kinase, ideally from a mammalian source, but not C5orf33 expressed in microbes, like *E. coli*.

This finding of human mitochondrial NAD kinase is deemed physiologically and medically important in relation to the so-called “mitochondrial diseases” such as chronic progressive external ophthalmoplegia, myoclonus epilepsy associated with ragged red fibers and mitochondrial myopathy, encephalopathy, lactic acidosis, and stroke-like episodes. Poly(P) has been noted in the mitochondria of mammalian cells.^87)^ The possibility has not necessarily been canceled that mitochondrial NAD kinase acts using poly(P) besides ATP. Why has mitochondrial ATP/poly(P)-NAD kinase not evolved to ATP-NAD kinase? Whether mitochondrial NAD kinase has merely been left behind by evolutionary change or that the vigorous respiratory environment in mitochondria has prevented it from evolving is yet to be determined. Although not convincing, it is also interesting to imagine that the ATP/poly(P)-NAD kinase gene in the human genome might come from mitochondria, which are believed to have originated from α-proteobacteria.

Nonetheless, a long-awaited NAD kinase in human mitochondria was finally found, wherein its involvement in the elimination of ROS was indicated. This is a critical discovery derived from the applied study of poly(P) and microbial NAD kinases. Recently, the roles of mitochondrial NAD kinase in physiological and pathological processes and the relationship between mitochondrial NAD kinase and the above-described diseases have been clarified, mainly through the analysis of functional impairment of mitochondrial NAD kinase gene.^88–91)^ The identification of the human mitochondrial NAD kinase undoubtedly provides a pivotal clue to the mechanism in NADPH production and the maintenance of redox balance in mammalian cells.

Following C5orf33 discovery, mitochondria-localized NAD kinase was confirmed in the liver.^92)^ The discovery of NAD kinase C5orf33 in human mitochondria and its physiological significance were highlighted by Ren Zhang, with an impressive title “MNADK, a long-awaited human mitochondrion-localized NAD kinase”.^93)^ Ren Zhang claimed that the HUGO Gene Nomenclature Committee named the mitochondrial NAD kinase gene “NADK2, mitochondrial” as a synonym.^93)^

## Concluding remarks

The novel bacterial NAD kinase, that is, ATP/poly(P)-NAD kinase, was predicted in a study on NADP^+^ production from NAD^+^ and ATP using ATP-NAD kinase.^14,94–96)^ When the NAD kinase reaction in *A. butyri* cell extracts (NAD^+^ + ATP → NADP^+^ + ADP) was coupled with ATP regeneration reaction catalyzed by poly(P) kinase (ADP + poly(P)_n_ → ATP + poly(P)_n−1_), a large amount of NADP^+^ was formed even in the absence of ATP. This suggests that the NAD kinase of *A. butyri* is poly(P)-dependent and catalyzes the following reaction: NAD^+^ + poly(P)_n_ → NADP^+^ + poly(P)_n−1_. The NAD kinase was analyzed and, in 1980, finally determined to be a novel ATP/poly(P)-NAD kinase that uses poly(P) besides ATP as phosphoryl donors.

Following this serendipitous discovery of poly(P)-dependent NAD kinase, in 2000, we were able to identify for the first time the gene encoding the NAD kinase in *M. tuberculosis* H37Rv^19)^ and, in 2012, the long-awaited human mitochondrial NAD kinase.^20)^ These pioneering results originated from the applied study on NADP^+^ production. We have spearheaded bioscience initiatives primarily related to microbial NAD kinases and paved the way for future research on NAD kinase and mitochondrial function.

In bacterial cells, poly(P) is described as a molecule functioning as energy and phosphate storage and in metabolic regulation of some energy-requiring processes, including roles in gene expression and stress response. These two functions of poly(P), *i.e.*, energy metabolism and physiological regulation, are possibly orchestrated by poly(P) kinase, which is a principal enzyme responsible for the reversible synthesis of poly(P).^4,6,15,16)^ Poly(P) is also found in various mammalian cell lines, tissues, and subcellular fractions including mitochondria.^97)^ Although poly(P) level in eukaryotic cells changes dynamically and rapidly in response to mitochondrial respiration and oxidative phosphorylation,^87)^ the ubiquity of poly(P) is indicative, as in the case of bacteria, of an assortment of functions in mammalian systems.

However, the enzyme responsible for poly(P) synthesis in mammalian cells remains to be elusive. DNA database search showed no obvious homology between bacterial poly(P) kinases and the mammalian genome, indicating that mammals lack a dedicated poly(P) kinase. Several pieces of evidence indicate that this polymer occurs through a mechanism involving the F_1_F_0_-ATP synthase using a proton gradient in mechanisms similar to ATP synthesis.^87)^ The detailed elucidation of the poly(P) synthetic process requires urgent attention to explain the function of poly(P)-dependent NAD kinase present in human mitochondria,^20)^ especially from the standpoint of the mechanism underlying the maintenance of intracellular balance among NAD^+^, NADP^+^, and their reduced forms.^81,98,99)^ Poly(P) research in humans and other higher eukaryotes has been restricted due to lacking suitable tools or enzymes for more comprehensive studies. The finding of ATP/poly(P)-NAD kinase (C5orf33 protein) in human mitochondria and other animals may provide new tools to investigate the function of poly(P) instead of the single-cell model system.

Regulation of NAD kinase activity by Ca^2+^/calmodulin is a crucial issue. In sea urchin eggs and human neutrophils, NAD kinase has been reported to be activated by Ca^2+^/calmodulin.^86,100)^ However, Ca^2+^/calmodulin has no detectable effect on recombinant human NAD kinase.^57)^ Alternatively, recombinant *A. thaliana* NAD kinases (NADK1, NADK2, and NADK3) are determined to be Ca^2+^/calmodulin-independent^36,56)^ and not activated by Ca^2+^/calmodulin, even though recombinant NADK2, which has a calmodulin-binding motif in its N-terminal, binds to calmodulin.^56)^ Thus, the results obtained on the regulation of NAD kinase activity by Ca^2+^/calmodulin are deemed controversial and unreliable. This may be due to the differences in recombinant and native NAD kinases. The use of other possible host organisms, *e.g.*, *Pichia pastoris*, but not *E. coli*, might allow successful expression of recombinant NAD kinases that respond to Ca^2+^/calmodulin like that of the native forms. Recent studies appear to have partially elucidated these problems. The study on the effect of Ca^2+^/calmodulin on human mitochondrial NAD kinase is significant since it directly reflects the NADPH concentration and ROS elimination activity of the mitochondria.

Although the data presented in this review are insufficient, analysis on the primary structures of ATP-NAD kinase and ATP/poly(P)-NAD kinase indicates the evolution process of NAD kinase, *i.e.*, from ATP/poly(P)-NAD kinase to ATP-NAD kinase. This changed the residues located behind the GGDG motifs in the order of GGDGT (ATP/poly(P)-NAD kinase) → GGDGS (ATP/poly(P)-NAD kinase) → GGDGN (ATP-NAD kinase)^62)^ (Fig. 3). The molecular sizes of the NAD kinase were constantly maintained during this evolution process. However, this feature of NAD kinase evolution contrasts with that of glucokinases, *i.e.*, ATP/poly(P)-glucokinase → ATP-glucokinase → ATP-hexokinase, through the evident increase in molecular sizes by adding flexible regions or domains.^101)^ The primary and tertiary structural studies of poly(P)-glucokinase^102)^ and poly(P)-NAD kinase, the latter of which has not been found, are indispensable for constructing precise evolution processes of the two kinases dependent on poly(P).

The information on the abovementioned evolutionary processes of NAD kinase makes it possible to create ATP/poly(P)-NAD kinase from ATP-NAD kinase^62,95)^ and NAD(H) kinase from NAD kinase^42)^ using a single amino acid substitution. By adding another unidentified structural determinant, ATP/poly(P)-NAD kinase could be further converted to poly(P)-NAD kinase, which is an ultimate ancestor of NAD kinase, which may have many suitable traits for application to industrial NADP^+^ production.^95)^ Although it is evident that this method is not universally applicable to any ATP-specific enzyme, this is the first example of successfully conferring the ability to use poly(P) on ATP-specific enzymes.^62)^

Some of the molecular mechanics, machinery, or biological systems in the past may be superior in potency and efficacy than those of the present. Although the title of this review represents the discovery of human mitochondrial NAD kinase, the excavation of functions being inherited in organisms from ancient times or its re-construction or both, which have been lost through natural selection in molecular evolution, are of special interest to me. These studies will thus contribute to the recreation and reproduction of the embryonic stage of cell development, in which self-dividing “droplets” evolved into protocells in a primordial soup containing poly(P) and other premature life compounds.^103)^

Judging from the close relationship between poly(P) and alginate, such as the co-regulation of poly(P) and alginate synthesis,^104)^ regulation of chemotaxis and motility by poly(P),^105)^ ability of flagellin (a principal component of bacterial flagellum) to bind alginate,^106)^ presumed existence of alginate-synthetic pathway in ancient bacteria,^107)^ presence of bacteria with an ingenious and unconventional mouth (super channel) to swallow alginate,^108)^ and formation of alginate-based biofilm^109)^ or Ca^2+^-alginate gels,^110)^ which provide a sequestered space, in which cells can grow, uronic acid, a component of alginate, might be one such compounds as with poly(P). In order to understand previous biological systems, we have no choice but to accumulate evidence regarding the traces of life, even if they were insignificant or far from the conditions of primitive earth.

## Figures and Tables

**Table 1.** Properties of NAD kinase

Organism		Abbreviated name	a.a.^1)^	Molecular structure	Acceptability^2)^		Ref.
					Poly(P)	NADH	
Procaryote							
Archaea	*Methanococcus jannaschii*	MJ0917	574	64 kDa × 4	+	+	46)
	*Pyrococcus horikoshii*	PH1074	277	37 kDa × 4	++	+	51)

Eubacteria							
Gram-positive	*Mycobacterium tuberculosis*	Ppnk	307	35 kDa × 4	+++	+	19), 42)
	*Myclococcus flavus*	Mfnk	362	34 kDa × 2	++	++	38), 42)
	*Bacillus subtilis*	bsu11610(NadF)	266	30 kDa × 2	+	nd^3)^	52)

Gram-negative	*Escherichia coli*	ecoNADK(YfjB)	292	30 kDa × 6	-	-	42), 47)
	*Sphingomonas* sp.	sphNADK(NadK)	298	32 kDa × 2	-	-	48)
	*Salmonella enterica*	styNADK	nd	nd	-	-	48)

Eucaryotes							
Yeast	*Saccharomyces cerevisiae*	Utr1p	530	60 kDa × 6	-	+	42), 50)
		Yef1p	495	60 kDa × 8	-	+	49)
		Pos5	414	46 kDa × nd	nd	+++	24), 52)

Plant	*Arabidopsis thaliana*	NADK1	524	58 kDa × nd	-	+++	56)
		NADK2	985	110 kDa × nd	-	nd	55)
		NADK3	317	38 kDa × 2	-	++++	36)

Human	*Homo sapiens*		446	49 kDa × 4	nd	(+)^4)^	57), 58)

1) a.a.: Number of amino acid residues.

2) Acceptability: activity as phosphoryl donor or acceptor; ++++, very high; +++, high; ++, moderate; +, low; -, none.

3) nd, unpublished or not determined.

4) Confirmed by thin layer chromatography.

This table was reproduced from S. Kawai and K. Murata (2008) Biosci. Biotechnol. Biochem. **72**, 919–930 (Ref. 63) with some modifications.
